# Antimicrobial Resistance in Sub-Saharan Africa: A Comprehensive Landscape Review

**DOI:** 10.4269/ajtmh.25-0035

**Published:** 2025-05-20

**Authors:** Valentina Totaro, Giacomo Guido, Sergio Cotugno, Elda De Vita, Muhammad Asaduzzaman, Giulia Patti, Francesco Vladimiro Segala, Giovanni Putoto, Luisa Frallonardo, Ferenc Balázs Farkas, Botond Lakatos, Nicola Veronese, Pietro Locantore, Francesco Di Gennaro, Annalisa Saracino

**Affiliations:** ^1^ Clinic of Infectious Diseases, Department of Precision and Regenerative Medicine and Ionian Area, University of Bari, Bari, Italy;; ^2^ Department of Community Medicine and Global Health, Institute of Health and Society, Faculty of Medicine, University of Oslo, Oslo, Norway;; ^3^ Operational Research Unit, Doctors with Africa CUAMM, Padua, Italy;; ^4^ Institute of Medical Microbiology, Faculty of Medicine, Semmelweis University, Budapest, Hungary;; ^5^ Pediatric Center, Semmelweis University, Budapest, Hungary;; ^6^ Deparment of Internal Medicine and Hematology, Departmental Group of Infectious Diseases, Semmelweis University, Budapest, Hungary;; ^7^ Faculty of Medicine, Saint Camillus International University of Health Sciences, Rome, Italy;; ^8^ Unit of Endocrinology, Department of Translational Medicine and Surgery, Università Cattolica del SacroCuore, Fondazione Policlinico “A. Gemelli” IRCCS, Rome, Italy

## Abstract

Antimicrobial resistance (AMR) is a critical health challenge in sub-Saharan Africa (SSA), driven by socioeconomic disparities, weak healthcare systems, and inadequate pharmaceutical regulation. This review examines AMR prevalence, drivers, and consequences in SSA, emphasizing the need for urgent interventions. A literature review was conducted using PubMed, Web of Science, Scopus, and Google Scholar, including studies published from January 2000 to June 2024. The focus was on AMR epidemiology, public health impacts, and interventions specific to SSA. High resistance rates were identified in *Escherichia coli*, *Klebsiella pneumoniae*, and *Staphylococcus aureus*. Key drivers include limited healthcare access; antibiotic misuse; poor surveillance; inadequate water, sanitation, and hygiene infrastructure; and poverty. AMR leads to increased mortality, prolonged hospital stays, and higher healthcare costs, with SSA projected to face 4.1 million AMR-related deaths annually by 2050 without action. Addressing AMR in SSA requires strengthening healthcare systems, expanding surveillance, enforcing pharmaceutical regulations, and enhancing education. International collaboration and funding are essential to mitigate AMR’s impacts and support progress toward universal health coverage and the Sustainable Development Goals.

## INTRODUCTION

The WHO defines antimicrobial resistance (AMR) as the ability of microorganisms—including bacteria, fungi, parasites, or viruses—to resist treatments that were previously effective against them. Recognized as one of the top 10 global health threats, AMR is projected to rival cancer as a leading cause of death by 2050, potentially accounting for 10 million deaths annually.^1^ Although AMR is a growing concern among fungi, mycobacteria, and parasites, the most critical threat arises from a group of bacteria collectively referred to as ESKAPEE pathogens (*Enterococcus faecium, Staphylococcus aureus, Klebsiella pneumoniae, Acinetobacter *(*A.*)* baumannii, Pseudomonas *(*P.*)* aeruginosa, Enterobacter* species, and *Escherichia *[*E.*] *coli*). These bacteria are predominantly associated with healthcare-related transmission and antimicrobial overuse. Hospitalization, in particular, is a well-established risk factor for AMR colonization and transmission, but a common misconception is that AMR stems solely from poor treatment management in hospitals. However, it is driven by a multitude of factors, including environmental stressors such as plastic pollution, which create conditions conducive to resistance in both human populations and the environment. Key contributors to AMR spread include the use of antibiotics in veterinary medicine and agriculture, as well as climate change and environmental contamination from healthcare, industrial, agricultural, and domestic waste.^2^^–^^4^

Table 1 outlines the primary drivers of AMR in the hospital setting.^5^ Although AMR is a universal challenge, its impact is disproportionately severe in fragile healthcare systems, such as those found in resource limited settings such as Africa and Asia.^6^ Sub-Saharan Africa (SSA) faces unique and significant challenges in combating AMR, which imposes a greater economic and health burden compared with other regions. In addition, we have observed profound disparity in the regional AMR surveillance within Africa as the majority of laboratories across Africa lack the capacity to effectively AMR testing.^7^ SSA embodies many of the problematic behaviors listed in Table 1.^8^ Most notably, SSA countries often lack national antibiotic stewardship programs, and weak regulatory frameworks have led to widespread misuse of antimicrobials in both healthcare facilities and agricultural settings.^9^ The financial constraints faced by low- and middle-income countries (LMICs) in SSA exacerbate the problem, driving reliance on substandard medications and resulting in suboptimal treatments.^10^ Limited resources also hinder the implementation of proper diagnostic tools, forcing physicians to rely on empirical treatments that are often inappropriate. This further complicates the tracking and monitoring of AMR, making it difficult to assess emerging resistance rates in specific pathogens.^8^^,^^9^ As a result, major drug-resistant pathogens such as extended-spectrum, beta-lactamase (ESBL)-producing bacteria, which represent a key driver of AMR-related mortality, are increasingly reported across SSA.^11^

**Table 1:** Healthcare behaviors/factors associated with AMR spread

Lack of personal protective equipment
Overcrowded wards with no possibility for contact isolation
Unavailable or poor diagnostic with improper use of antibiotics
Excessive use of antibiotics from the physicians
Higher need for surgery (especially in armed conflicts)
Hemato–oncological and cancer treatment facilities (with a higher prevalence of antibiotic prophylaxis)
Hospitals overwhelmed with water scarcity resulting in inadequate hand hygiene practices
Lack of equipment for cleaning and sterilization
Inappropriateness of antibiotic prescription
Limited education of health workers in antimicrobial stewardship
Substandard and falsified medicines
Suboptimal regulation on perioperative antibiotic prophylaxis
Antimicrobials available without prescription
Low-resource settings and underinvestment in healthcare

AMR = antimicrobial resistance.

Additionally, SSA is disproportionately impacted by climate change, which exacerbates poor sanitary conditions and degrades water quality. These environmental factors act as reservoirs for bacterial proliferation and contribute to the spread of AMR.^12^^,^^13^

**Figure 1.**
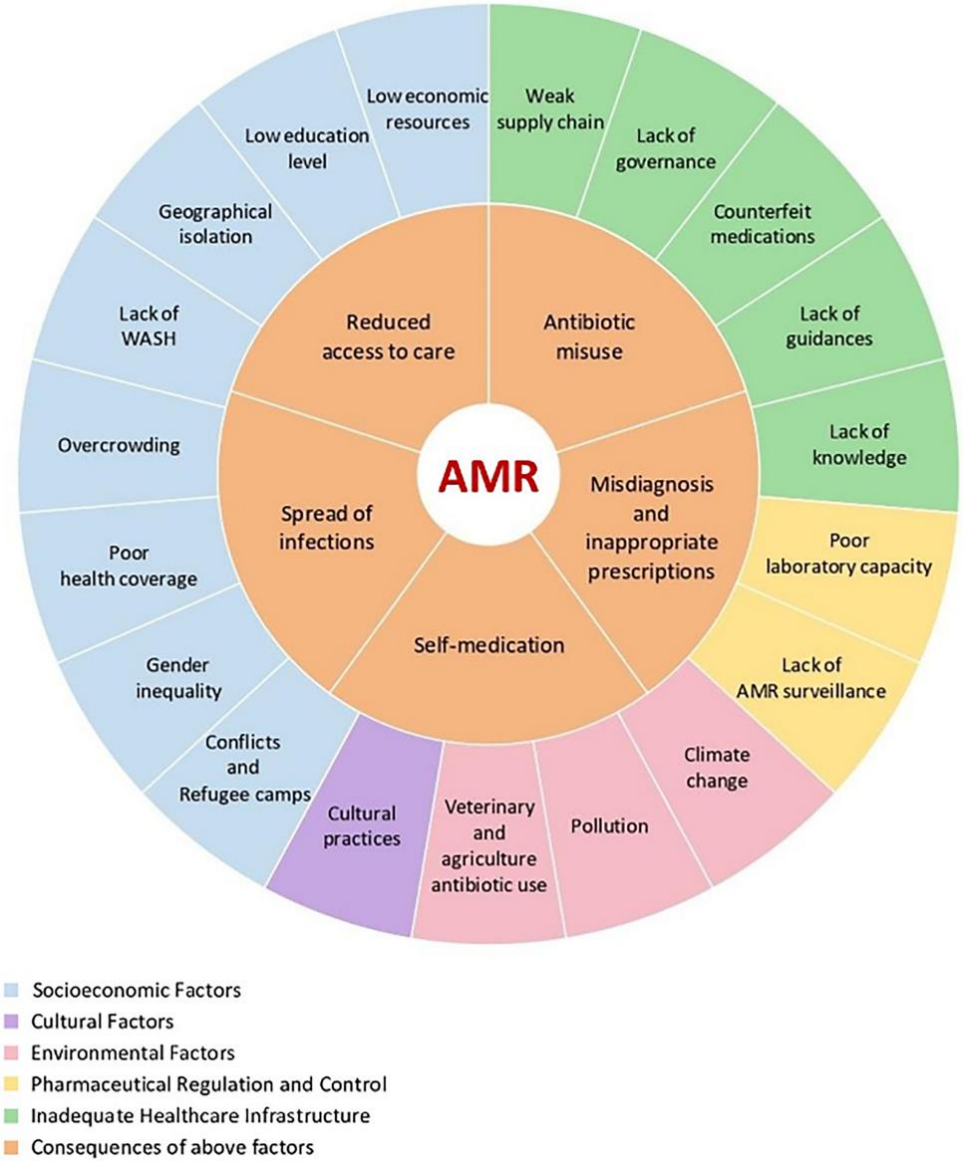
The complexity of drivers of antimicrobial resistance (AMR) in sub-Saharan Africa.

Addressing AMR in SSA requires a comprehensive, One Health approach that integrates public awareness, education on the responsible use of antibiotics, stronger regulatory frameworks, and improved healthcare infrastructure.^14^^–^^16^ Without such measures, SSA will continue to face escalating threats from AMR. The primary objective of this review is to examine the prevalence and impact of AMR in SSA. This includes a comprehensive analysis of the current state of AMR, encompassing its distribution and its effects on public health. Additionally, the review aims to identify the key drivers of AMR in SSA, including environmental factors, healthcare practices, and socioeconomic determinants, as well as to evaluate the effectiveness of existing strategies designed to mitigate its impact (Figure 1). A particular focus is placed on ensuring sustainable approaches to addressing this critical health challenge. Given the complexity of the topic, the scope of this review is limited to AMR in pathogens associated with healthcare settings and commonly used antimicrobials. The discussion of AMR in mycobacteria, viruses, malaria, and other parasites is deferred to other specific studies.

## MATERIALS AND METHODS

### Data sources and search strategy.

This literature review used a comprehensive search strategy using multiple academic databases, including PubMed, Web of Science, Scopus, and Google Scholar. The search incorporated a combination of keywords and Medical Subject Headings terms, such as AMR, SSA, “epidemiology,” “public health impact,” and “causes of AMR.” To reflect the dominant languages in the region’s scientific literature, the search was restricted to articles published in English and French. The inclusion criteria were carefully defined to ensure the selection of relevant and high-quality studies. Publications considered for the review were those published between January 2000 and June 2024, focusing on research conducted in SSA. Both peer-reviewed articles and reports from health organizations and governments were included. To be eligible, studies had to address at least one of the following topics: the epidemiology of AMR, the public health impact of AMR, or the risk factors of AMR in SSA.

## RESULTS

### Prevalence of AMR in sub-Saharan Africa.

The prevalence of AMR in SSA represents a growing public health crisis, with significant variability observed across different countries in the region.^17^ Although these statistics are alarming, they fail to capture the full scope of the issue because of substantial data gaps across the continent. A systematic review by Tadesse et al. in 2017 revealed that data were unavailable for 42.6% of SSA countries, with Western Africa being particularly underrepresented.^18^ The primary reasons for this lack of surveillance included inadequate laboratory diagnostic capacities, insufficient infrastructure, and the unpreparedness of national health ministries to implement AMR monitoring systems. Since 2017, some improvements have been noted, particularly in West Africa, where the number of national AMR monitoring centers has doubled.^19^ This progress has been facilitated, in part, by the WHO’s Global AMR and Use Surveillance System (GLASS), launched in 2015 to collect AMR data and assess their impact on human health globally.^20^ Despite these advancements, the burden of AMR in SSA continues to rise, emphasizing the urgent need for a One Health approach and the implementation of comprehensive interventions starting with the strengthening of surveillance.

### Bacterial infections.

The increasing prevalence of AMR among bacterial pathogens in SSA has become a major public health concern. Studies report a rising frequency of multidrug-resistant organisms, particularly *E. coli*, *Klebsiella pneumoniae*, and *Staphylococcus aureus.*^18^ Resistance to widely used oral antibiotics, such as amoxicillin and ciprofloxacin, is becoming increasingly common, significantly limiting effective treatment options.^21^ The situation is especially concerning in vulnerable populations, such as maternal and pediatric groups, where resistance rates among common pathogens are particularly high.^22^^,^^23^

### Methicillin-resistant *Staphylococcus aureus* (MRSA).

On the WHO’s critical list, MRSA remains a priority pathogen because of its widespread prevalence and significant impact on public health.^24^ In high-income countries, MRSA, alongside *E. coli*, accounts for nearly 50% of deaths attributable to AMR.^25^ However, in LMICs, particularly in SSA, the prevalence of MRSA is alarmingly high. Sub-Saharan and central Africa exhibit MRSA infection rates of approximately 40%, compared with lower rates observed in South Africa. Colonization rates of MRSA in SSA vary between 5% and 23%, with the highest rates seen in populations with chronic medical conditions.^26^ Unfortunately, data from SSA on MRSA prevalence are sparse in global systematic reviews, largely because of limited surveillance systems, underreporting, and insufficient laboratory capacity. Consequently, estimates of MRSA prevalence often rely on isolated studies or specific hospital reports, which may not accurately reflect the broader regional burden. Compounding the problem is the limited availability and accessibility of anti-MRSA treatments in SSA such as vancomycin, linezolid, and fifth-generation cephalosporins (e.g., ceftaroline) or daptomycin.^27^^,^^28^

### Enterobacterales.

In SSA, AMR among *Enterobacterales* represents a significant public health threat, as these pathogens are major contributors to sepsis, urinary tract infections, and hospital-acquired pneumonia. Recent systematic reviews indicate that ESBL-producing *Klebsiella pneumoniae* and *E. coli* exceed 40% prevalence in certain SSA countries.^29^ Resistance rates in pediatric infections caused by *Enterobacterales* surpass 40%, with similar figures observed in bloodstream infections and urine samples.^23^ The southern regions of SSA report comparatively lower prevalence rates, whereas West and East Africa are the most affected, as a possible result of lower antibiotic consumption because of regional economic disparities.

Carbapenemase-producing bacteria, including those expressing New Delhi metallo-beta-lactamase and oxacillinase-48, pose an even greater concern, highlighting the global impact of carbapenem resistance.^30^ Although carbapenems, such as meropenem, have been available in SSA since the early 2000s, their high cost and slow approval processes have limited their use. Despite their relatively restricted availability, carbapenem-resistant *Enterobacterales* are already a significant burden in African healthcare settings.

### *Shigella, Salmonella,* and cholera.

*Shigella* species are a leading cause of diarrheal disease in SSA, with a pooled prevalence of 5.9%.^31^ They account for 13% of all diarrhea-associated deaths in the region.^32^ Resistance to cotrimoxazole and ampicillin is widespread, prompting the adoption of ciprofloxacin and third-generation cephalosporins as first-line treatments. However, emerging evidence suggests rising resistance to these therapies, with ciprofloxacin resistance reaching 10% in some regions of SSA.^31^

*Salmonella* spp. accounts for approximately 22% of all diarrhea-related illnesses globally, with mortality rates reaching 20% among vulnerable populations in SSA.^33^^,^^34^ Standard treatments for salmonellosis include ciprofloxacin, third-generation cephalosporins (e.g., ceftriaxone), or azithromycin, depending on local resistance patterns.^35^^,^^36^ Nonetheless, resistance to these antibiotics is increasing, particularly in resource-limited settings. In SSA, resistance rates for ciprofloxacin and third-generation cephalosporins are estimated at approximately 10%, complicating the effective management of *Salmonella* infections.^37^

Data on *Salmonella* and *Shigella* resistance reported through the GLASS program should be interpreted cautiously because of limited sample sizes. Despite these limitations, the growing resistance to ciprofloxacin—a widely used and practical therapy—remains a significant concern.^35^

Cholera, caused by *Vibrio *(*V.*)* cholerae*, is another severe diarrheal disease in SSA. Although cholera is not officially recognized by the WHO as a major AMR concern, multidrug-resistant strains of *V. cholerae* O1/O139, responsible for epidemic outbreaks, have been documented.^38^ Resistance to first-line therapies such as tetracyclines has been reported, alongside beta-lactam resistance, with prevalence rates reaching up to 20%.^39^^,^^40^ However, most available data on cholera resistance originate from Asia, with limited information specific to SSA.^38^

Children under 5 years old and internally displaced persons are particularly vulnerable to *Salmonella* and cholera infections because of malnutrition, poor sanitation, and limited access to healthcare.^41^^–^^43^ Recent outbreaks in SSA have disproportionately affected these groups, highlighting the urgent need for targeted interventions and improved disease surveillance.^44^^,^^45^

### Non-fermenting gram-negative bacteria.

Non-fermenting gram-negative bacteria, including *P. aeruginosa* and *A. baumannii*, are opportunistic pathogens frequently associated with healthcare settings. These bacteria are particularly problematic in intensive care units. However, data from SSA remain sparse because of inconsistent diagnostic capabilities and heterogeneous healthcare infrastructure.^46^ Recent systematic reviews estimate that carbapenem-resistant *P. aeruginosa *and *A. baumannii* account for 8% and 20% of isolates, respectively.^47^

In addition, postsurgical infections represent a critical yet often overlooked driver of AMR in SSA, particularly in conflict-affected regions where surgical interventions are more frequent and infection control measures are inadequate.^48^^–^^50^ The high prevalence of surgical site infections, compounded by limited access to sterilization equipment, appropriate antibiotic prophylaxis, and postoperative care, contributes significantly to AMR. Studies have shown that AMR in postsurgical infections is associated with increased morbidity and mortality, further straining already fragile healthcare systems. Addressing this challenge requires targeted interventions, including improved perioperative antibiotic stewardship and enhanced infection prevention protocols in resource-limited settings.^49^^–^^51^

### Vancomycin-resistant enterococci.

Enterococci, particularly *Enterococcus faecium* and *Enterococcus faecalis,* have evolved from being gut commensals into significant hospital-associated pathogens because of rising antibiotic resistance.^52^ In SSA, the pool prevalence of vancomycin-resistant enterococci (VRE) is reported at 10.2%, although most studies focus on Southern Africa.^53^ Studies from specific countries report even higher rates in some settings, highlighting significant regional variation.^54^^,^^55^ These findings underscore the urgent need for enhanced surveillance and infection control strategies. Similar to MRSA, the limited availability and high cost of effective treatments in SSA present significant barriers to controlling VRE infections.^56^

#### Fungal infections.

Invasive fungal infections (IFI) are gaining prominence because of their rising incidence and associated antifungal resistance, prompting the WHO to release a fungal priority pathogen list.^57^ Among the critical priority pathogens identified are *Cryptococcus neoformans*, *Candida *(*C.*)* auris*, *C. albicans*, and *Aspergillus fumigatus*. IFI are generally linked to immunosuppression and healthcare-associated infections. Azoles are the first-line treatments for aspergillosis and candidiasis; however, resistance to these antifungals is increasing. For instance, recent studies show that resistance in invasive infections caused by *C. albicans* has risen from 20% to 60%.^58^ As a result, physicians are increasingly relying on second-line therapies, such as amphotericin B and echinocandins, which are significantly more expensive and less accessible in LMICs. In SSA, data on IFI remain sparse, with South Africa being the primary source of reported findings.^59^ The diagnosis of IFI is complicated by the low sensitivity of blood cultures, necessitating the use of non-culture-based assays, which remain challenging to implement in resource-limited settings. These diagnostic gaps are especially concerning given the high prevalence of HIV in SSA, a major risk factor for IFI.^60^ Candidiasis caused by *C. albicans* and *non-albicans C*. spp. in SSA is characterized by alarmingly high rates of fluconazole resistance, reaching up to 100% in some cases.^61^ Although voriconazole remains effective,^62^ emerging concerns about potential resistance highlight the need for careful monitoring. However, many studies do not differentiate between pathogenic infections and colonization, making resistance estimates less reliable.^63^^,^^64^ As a result, echinocandins are now recommended as first-line treatments for *C. *infections, despite their limited availability in only 12 SSA countries.^65^

Cryptococcosis, a severe disease affecting people living with HIV and caused by *Cryptococcus neoformans*, is typically treated with amphotericin B and flucytosine as first-line therapies. However, the prolonged duration and toxicity of these treatments often result in adverse effects, such as renal impairment, necessitating a switch to fluconazole. Moreover, after the induction treatment phase, a long-term maintenance (6–12 months) phase with fluconazole is needed.^66^ SSA accounts for 73% of global cryptococcosis cases, with fluconazole resistance reported in 43.6% of isolates.^67^ However, most of these data come from South African cohorts. Resistance to fluconazole is often associated with poor adherence to treatment regimens. In vitro studies suggest that prolonged suppressive therapy with fluconazole correlates with lower resistance rates, underscoring the importance of adherence.^68^

#### Other pathogens of concern.

##### *Streptococcus* spp.

Group A Streptococci (GAS), group B Streptococci (GBS), and *Streptococcus *(*S.*)* pneumoniae* are pathogens of concern recently prioritized by the WHO. GAS is a major contributor to rheumatic heart disease, which causes over 300,000 deaths annually, predominantly in LMICs.^69^ Reports of macrolide-resistant GAS are emerging, with speculation linking the broader spread of resistance to the misuse of azithromycin during the coronavirus disease 2019 pandemic.^70^ Vulnerable populations, particularly infants and the elderly, face substantial impacts, although specific data on macrolide-resistant GAS in SSA remain unavailable.^27^

GBS are associated with a high risk of neonatal and infant sepsis, meningitis, maternal sepsis, and preterm delivery. As a result, many high-income countries implement screening programs during pregnancy and intrapartum prophylaxis. However, in LMICs, these measures are often unfeasible because of limited resources. SSA accounts for an estimated 54% of invasive GBS cases globally, making it critical to monitor the emergence of resistance. Current data on penicillin-resistant GBS in SSA are sparse, typically derived from single-center studies. Reported rates include 13.8% penicillin-resistant GBS and 17.2% ceftriaxone-resistant GBS.^71^ Despite WHO’s release of a roadmap for GBS vaccine development in 2017, only three candidate vaccines have reached phase II trials as of now.^72^ In contrast, vaccination against *S. pneumoniae* has been available for decades, although coverage remains inconsistent across regions. As of 2023, the estimated coverage for the third dose of the pneumococcal conjugate vaccine in the WHO African region was only 48%.^73^ These disparities, coupled with the emergence of macrolide resistance, pose significant challenges. Limited data from SSA suggest alarming resistance rates, with one study reporting a 33% prevalence of macrolide-resistant *S. pneumoniae* in pediatric populations.^74^^–^^76^

##### Neisseria gonorrheae.

*Neisseria* gonorrhoeae is the only sexually transmitted infection pathogen recognized by the WHO as a critical concern for AMR. Resistance to fluoroquinolones in SSA is alarmingly high, with reported rates reaching approximately 53%. Ceftriaxone resistance, a key challenge in gonorrhea treatment, shows significant regional variation across SSA, ranging from 4% to 48%.^77^ In addition to resistance to fluoroquinolones and cephalosporins, increasing resistance to macrolides, such as azithromycin, warrants close monitoring and underscores the urgent need for novel therapeutic strategies.^78^

#### Factors contributing to AMR in sub-Saharan Africa.

##### Socioeconomic factors: poverty, education, and urbanization.

Socioeconomic factors play a significant role in AMR emergence, spread, and impact, particularly in LMICs.^79^ In SSA, access to healthcare is severely limited by poor health coverage, particularly in rural areas, where the availability of essential health services ranges from 20% to 59% in most countries, exceeding 60% only in Namibia and South Africa.^80^^,^^81^ Rural areas also face a lack of clean water, electricity, and adequate sanitation, further compromising the quality of care.^82^^,^^83^ More than half of SSA’s population lives in geographically isolated rural settlements,^84^ where long travel times to healthcare facilities, understaffing, and shortages of essential medical supplies are common.^83^^–^^86^ Consequently, marginalized populations experience poor health outcomes,^87^ with inadequate treatment leading to antibiotic misuse and the emergence of multidrug-resistant bacteria.^88^^–^^90^

Economic barriers exacerbate these challenges. In SSA, high out-of-pocket health expenditures deter people from seeking care,^91^ whereas the private healthcare sector, often the only viable option,^92^ remains unaffordable for many.^93^

The absence of free-of-charge healthcare services in many African countries significantly impacts antibiotic adherence, as financial constraints often force patients to discontinue treatment prematurely. This contributes to suboptimal antibiotic use, increasing the risk of incomplete pathogen eradication and fostering the emergence of AMR.^92^^–^^94^ Nearly half of the region’s population lives in poverty,^94^ restricting access to essential services such as healthcare, education, and sanitation.^95^^,^^96^

Poverty limits access to healthcare, leading to higher rates of self-medication and the use of substandard or counterfeit antibiotics, often purchased from informal markets. Additionally, high out-of-pocket healthcare expenditures force many individuals to forgo proper medical consultation, further contributing to the misuse of antibiotics.^97^^–^^99^

This perpetuates cycles of poverty and illness, with behaviors such as incomplete antibiotic courses, self-medication, and reliance on informal healthcare systems driving resistance.

Urban overcrowding and poor sanitation also contribute significantly to AMR. These conditions facilitate the spread of infectious diseases, including oro–fecal infections (e.g., cholera, *Klebsiella*, and *E. coli*) and airborne diseases (e.g., tuberculosis and *S. pneumoniae*), leading to excessive and often inappropriate antibiotic use.^88^^–^^90^ Furthermore, in an overcrowded environment, the risk of resistance gene dissemination is elevated.^100^^–^^102^

##### Healthcare barriers: infrastructure and accessibility.

Access to healthcare in SSA is hindered by a weak healthcare infrastructure, inadequate laboratory capacity, and limited surveillance systems. Many laboratories lack the equipment and reagents necessary for microbiological testing, resulting in misdiagnoses and the overuse of broad-spectrum antibiotics.^103^^–^^104^ Only 20 of 47 SSA countries contribute data to the WHO’s GLASS system, underscoring the fragmented nature of AMR surveillance.^105^ The African Society for Laboratory Medicine reports that only 1.3% of laboratories in SSA perform microbiological tests, with many providing incomplete clinical and treatment data.^106^ Furthermore, healthcare facilities often experience supply chain disruptions, leading to the use of substandard antibiotics or broad-spectrum antimicrobials, both of which exacerbate resistance.^107^^–^^109^

##### Cultural practices and traditional medicine.

Cultural factors also influence AMR development in SSA. Traditional medicine remains widely used, particularly among low-income and rural populations, where it is often more accessible and affordable than formal healthcare.^110^^,^^111^ However, the unregulated use of traditional remedies increases the risk of misdiagnosis, adverse reactions, and ineffective treatment, particularly when practices are not communicated to healthcare professionals.^112^^,^^113^

Conversely, traditional medicine offers potential in the fight against AMR. Plant-derived phytochemicals have demonstrated antibacterial properties and could serve as alternatives to synthetic antibiotics, reducing selective pressure on existing treatments.^113^^,^^114^

##### Environmental and agricultural contributions to AMR.

The misuse of antibiotics in veterinary medicine and agriculture is a major driver of AMR. In SSA, antibiotics are frequently used for growth promotion in livestock and crops, with tetracycline and streptomycin being the most common.^115^^,^^116^ This practice facilitates the transfer of resistant bacteria and genes through animal waste, contaminating water, food, and the broader environment.^117^^,^^118^

Plastic pollution is another emerging concern. Plastics can harbor resistant bacteria and act as reservoirs for AMR genes.^119^ Inadequate disposal of medical, agricultural, and industrial waste further exacerbates the accumulation of antibiotics and resistant bacteria in the environment.^116^^,^^117^

##### Pharmaceutical regulation and counterfeit medications.

Inappropriate antibiotic prescriptions and the widespread circulation of substandard medications are major contributors to AMR in SSA. A 2023 review reported alarmingly high rates of inappropriate antibiotic use in hospitals, largely driven by limited access to microbiological testing and an overreliance on empirical treatments.^120^^,^^121^ In addition, counterfeit and substandard antibiotics remain a significant challenge, with an estimated prevalence of 18.7% across Africa.^121^^,^^122^

Weak regulatory frameworks further exacerbate this issue, allowing the production and distribution of poor-quality drugs that not only fail to treat infections effectively, but also fuel resistance.^123^^,^^124^ Strengthening governance, enhancing quality control mechanisms, and enforcing stricter regulatory oversight are essential steps to curb the proliferation of counterfeit medications and mitigate their impact on AMR.

##### Dysfunctional health system, self-medication with antibiotics, and over-the-counter drug selling.

Inadequate public facilities, overburdened healthcare providers, limited diagnostic services, mistrust of healthcare professionals, and blurred distinctions between qualified and unqualified practitioners all contribute to the prevalence of self-medication with antibiotics. Unregulated antibiotic sales without prescriptions, unregistered pharmacies operating outside legal frameworks, and drug sellers dispensing medications without proper training are the major problems. Furthermore, insufficient oversight of antibiotic prescriptions in both formal and informal healthcare settings has fostered a lack of accountability among healthcare providers, including licensed physicians. Studies from LMICs in SSA underscore systemic health challenges that drive individuals to seek care from unqualified providers, as well as weak law enforcement that enables the widespread availability of over-the-counter antibiotics, exacerbating misuse.^124^^–^^126^

##### Gender inequality.

Gender disparities in SSA affect AMR in multiple ways. Women face additional hygiene challenges because of limited access to water, sanitation, and hygiene (WASH) systems, which increase their susceptibility to infections and reliance on antibiotics.^117^ Biological factors, cultural norms, and limited financial and educational opportunities further hinder women’s access to healthcare.^127^^,^^128^

Women and children represent particularly vulnerable populations in the context of AMR. Women often face barriers to healthcare access, gender-based disparities in health education, and frequent antibiotic exposure during pregnancy and childbirth, which may contribute to AMR risk. Similarly, children experience higher infection rates and are disproportionately affected by the limited availability of pediatric formulations for second-line antibiotics, often leading to suboptimal treatments.^129^^–^^133^

##### Refugee camps, conflicts, and climate change.

Political instability and climate change exacerbate AMR by disrupting healthcare systems and worsening living conditions. Refugee camps are particularly vulnerable because of overcrowding, poor sanitation, and limited access to medical care.^134^^–^^136^ Conflicts further degrade infrastructure and supply chains, leading to untreated or improperly treated infections. Rising temperatures associated with climate change also promote bacterial growth, potentially accelerating the spread of resistance.^137^^–^^140^

## IMPACT OF AMR

### Public health consequences.

The Drug Resistance Index score for SSA is alarmingly high, underscoring the out-of-control nature of AMR. This index reflects both the declining efficacy of antibiotics and the extent of their misuse.^125^ AMR adversely affects health systems by driving up morbidity, mortality, and healthcare costs, particularly in LMICs, where resources are often insufficient to combat the crisis.^140^^,^^141^ Africa has the highest global mortality rate from AMR infections. In 2019 alone, bacterial AMR was associated with an estimated 1.05 million deaths in the WHO African region, with 250,000 deaths directly attributable to resistant infections. The majority of these fatalities occurred in western SSA.^128^ Bloodstream infections, intra-abdominal infections, tuberculosis, and lower respiratory and thoracic infections were the leading causes of AMR-related mortality. The most common pathogen-drug combinations contributing to these deaths were MRSA and third-generation cephalosporin-resistant *Klebsiella pneumoniae*.

AMR also significantly increases the burden on health systems. Resistant infections result in longer hospital stays, more expensive treatments, and greater use of intensive care resources, including mechanical ventilation and invasive devices.^142^^,^^143^ In LMICs, where intensive care and costly treatments are less accessible, AMR is a major driver of fatalities.^144^^–^^146^ A retrospective study conducted in Senegal revealed that patients with ESBL-producing *Enterobacterales* infections had hospital stays more than 7 days longer and mortality rates twice as high compared with those with non-ESBL-producing infections.^147^

Future projections are equally concerning. Without immediate intervention, global deaths attributable to AMR are expected to rise by 69.6%, and AMR-associated deaths by 67.0%, by 2050. This increase would result in 4.1 million deaths in Africa alone.^148^ Proactive measures, such as infection prevention programs, expanded WASH infrastructure, and widespread vaccination, could prevent 18% of AMR-related deaths in LMICs annually.^149^

The growing threat of AMR could also reverse progress in modern medicine. Once-manageable infections may again become life-threatening, making surgeries like organ transplants or hip replacements exceedingly risky. Vulnerable populations, including cancer patients, the immunocompromised, and those requiring surgical interventions, face heightened risks from emerging drug-resistant bacterial strains.^150^^,^^151^ Historically rare infections could reemerge as antibiotics become increasingly ineffective.

#### Economic consequences.

The economic impact of AMR is equally severe, with projections indicating a potential reduction in global gross domestic product (GDP) by 2–3.5% by 2050, translating to an economic loss of $60–100 trillion.^152^ The World Bank estimates that AMR-related healthcare costs could reach $1 trillion by 2050.^1^ AMR increases healthcare expenses by prolonging hospital stays, necessitating more expensive drugs, requiring isolation measures, and increasing the demand for diagnostic services.^153^

The rising mortality from AMR also leads to workforce losses, reducing overall productivity, and economic output.^154^ For LMICs, where health systems and populations are already vulnerable, these costs are particularly unsustainable. The economic burden threatens progress toward universal health coverage and the Sustainable Development Goals, pushing millions into extreme poverty.^155^

Forecasts under a high-AMR scenario—where antimicrobials lose their effectiveness entirely—suggest that LMICs could experience GDP losses exceeding 5% and that up to 28 million people could fall into poverty by 2050, with the majority in developing countries.^156^ The global trade and livestock sectors would also suffer significant losses, further amplifying the economic impact.^157^^,^^158^

## CONCLUSION

SSA is facing a rise in AMR and its detrimental impact. Our review shows a prevalence of MRSA and ESBL-producing *Enterobacterales* of around 40% in SSA, which are globally recognized as priority resistant pathogens. There was also a high prevalence of fluconazole-resistant candidiasis and cryptococcosis, conditions that primarily affect people living with HIV, who are numerous in SSA. Finally, an increase in resistance was detected in infections caused by *Shigella*, *Salmonella* spp., and *V. cholerae*, leading causes of diarrheal diseases and high mortality.

Poverty and a dysfunctional healthcare system are likely leading factors in the emergence and spread of AMR in SSA. In addition, environmental factors such as the expansion of intensive farming and livestock production in SSA, along with the associated overuse of antibiotics, global warming, conflicts, and climate disasters, play a significant role by further impacting an already fragile socio-health context. Combating AMR necessitates international cooperation and targeted support for low-income regions such as SSA. Addressing AMR in SSA requires a multifaceted approach that integrates global health strategies with region-specific interventions. Global health organizations, donor agencies, and international governments must actively contribute by providing financial resources, technical expertise, and logistical assistance to address this growing crisis. Strengthening national action plans, investing in antimicrobial stewardship programs, and expanding laboratory capacity are essential steps. Effectively tackling AMR in SSA requires a comprehensive, multifaceted approach that addresses the root causes, including socioeconomic disparities, healthcare system weaknesses, and cultural practices. Strengthening healthcare infrastructure, enforcing robust pharmaceutical regulations, expanding surveillance systems, and promoting education and public awareness are critical components of a successful strategy to curb the rise of AMR. Moreover, mitigating the economic and public health consequences of AMR should be prioritized to prevent further destabilization of SSA’s already fragile healthcare systems. Without immediate and coordinated action, the unchecked spread of AMR threatens to reverse decades of progress in global health, disproportionately affecting vulnerable populations and deepening inequalities in access to care.
